# Cellular responses to Staphylococcus aureus alpha-toxin are associated with clinical outcomes in chronic rhinosinusitis with nasal polyps

**DOI:** 10.1186/2045-7022-3-S2-P25

**Published:** 2013-07-16

**Authors:** Mitushiro Okano, Takenori Haruna, Yasuyuki Noyama, Misato Hirai, Kazunori Nishizaki, Tazuko Fujiwara

**Affiliations:** 1 Okayama University Graduate School of Medicine, Dentistry and Pharmaceutical Sciences, Okayama, Japan; 2 Okayama University Graduate School of Medicine, Dentistry and Pharmaceutical Sciences, Otolaryngology-Head & Neck Surgery, Okayama, Japan; 3 Okayama Saiseikai General Hospital, Otolaryngology-Head & Neck Surgery, Okayama, Japan

## Background

In contrast to Staphylococcus aureus-derived superantigenic exotoxins, the role of non-superantigenic exotoxins in the pathogenesis of chronic rhinosinusitis (CRS) remains obscure.

## Objective

We sought to characterize S. aureus alpha-toxin--induced Th1-, Th2-, Th17-, and Treg-associated cellular responses in CRS with nasal polyps (CRSwNP).

## Method

Dispersed nasal polyp cells (DNPCs) and dispersed uncinate tissue cells (DUTCs) were prepared from patients with CRS with and without nasal polyps, respectively. Cells were incubated with various concentrations of alpha-toxin or staphylococcal enterotoxin B (SEB) and then the levels of IL-5, IL-13, IFN-gamma, IL-17A, and IL-10 in the cell supernatants were determined. The effect of blocking the COX pathway and neutralizing HLA-DR and ICAM-1 was examined. The pathophysiological significance of alpha-toxin--induced cytokine production was also determined.

## Results

DNPCs produced substantial amounts of IL-5, IL-13, IFN-gamma, IL-17A, and IL-10 in response to alpha-toxin. Cytokine production was higher in DNPCs than in DUTCs. The potency of alpha-toxin in stimulating IL-5, IL-13, and IL-10 production was comparable to that of SEB. Neutralization of HLA-DR and ICAM-1 suppressed cytokine production. Inhibition of the COX pathway increased and decreased alpha-toxin--induced production of IL-5/IL-13 and IL-17A/IL-10, respectively. Alpha-toxin--induced IFN-gamma, IL-17A, and IL-10 production negatively correlated with the degree of eosinophil infiltration into nasal polyps. Furthermore, alpha-toxin--induced IL-10 production correlated negatively with postoperative CT score and positively with radiological improvement assessed 6 months after sinus surgery.

## Conclusions

In addition to S. aureus-derived superantigens, non-superantigenic alpha-toxin can provoke cellular responses in nasal polyps. These responses, especially failure to synthesize IL-10, regulate the pathophysiology of CRSwNP, including nasal polyp formation and postoperative outcome.

